# School-based mental health literacy training shifts the quantity and quality of referrals to tertiary child and adolescent mental health services: A Western Canada regional study

**DOI:** 10.1371/journal.pone.0277695

**Published:** 2022-11-15

**Authors:** Andrew Baxter, Yifeng Wei, Stan Kutcher, David Cawthorpe

**Affiliations:** 1 School-Based Mental Health Team, Alberta Health Services, Calgary, Alberta, Canada; 2 Department of Psychiatry, Faculty of Medicine and Dentistry, The University of Alberta, Edmonton, Alberta, Canada; 3 Department of Psychiatry, Faculty of Medicine, Dalhousie University, Halifax, Nova Scotia, Canada; 4 Departments of Psychiatry and Community Health Sciences, Institute for Child and Maternal Health, Faculty of Medicine, The University of Calgary, Alberta, Canada; University of South Australia, AUSTRALIA

## Abstract

**Background:**

We aimed to improve mental health referral quality of young people by helping educators build capacity for early identification of youth at risk of mental illness and facilitate referrals between the education and health systems.

**Methods:**

We applied the Go-To Educator mental health literacy training for early identification, triage and support in 208 schools in Calgary, Alberta between 2013 and 2016. Students presenting to mental health services during this time were compared on a number of clinical, system, and demographic variables, based on the training status of the school (untrained schools; before and after training schools), using retrospective cohort design. Based on clinical and system data, bivariate and multivariable logistic regression analysis were employed to compare the three school status domains.

**Results:**

After training, referrals differed significantly from control and pre-training schools. Students presenting to services from these schools were younger, from single parent families; were referred more because of adjustment and learning/attention problems; had complex social/family issues; thought disturbances, and harmful behavior/thoughts towards others. While they waited longer to be admitted they stayed longer in services; had more provisional comorbid diagnoses and demonstrated positive treatment outcomes.

**Conclusions:**

The Go-To Educator training may be an effective intervention helping educators identify students at risk of mental disorders and in substantial need of mental health services, demonstrating improved linkages between education and health sectors.

## Introduction

The need to effectively improve access to care for youth with mental disorders is a global challenge. The World Health Organization has reported that mental disorders comprise the largest single category of medical disability in young people [1, 2]. Adolescence has been identified as a critical period for the onset of the majority of psychiatric illnesses [3]. Left untreated, mental disorders increase risk for numerous negative short- and long-term outcomes such as suicide, unemployment, criminal activity and poor school performance [4]. While mental disorders in young people can be effectively treated, many who need care do not receive it [5]. There are numerous barriers facing youth trying to access mental health services including lack of identification of illness by self or others, stigma, lack of knowledge about mental health, lack of funding, wait times, parental involvement, the youth’s geographic location and fragmentation of services [6–10]. Some of these barriers can be addressed by promoting early identification of youth with mental disorders and enhancing access to care in the school setting [11–14]. With most youth spending extensive periods of time at school, educators and other school personnel are well positioned to identify those who may be showing signs of mental illness. While there is substantial evidence that providing educators with mental health literacy training impacts on student’s knowledge and stigma reduction [12, 15–21], there is little—if any—research that measures the impacts of these school mental health literacy-based interventions on referrals of students from schools to mental health services. This study addresses that gap employing third party blinded data source characterizing the clinical profile of referrals from schools and other sources to a regional publicly funded child and adolescent mental service.

As mental health literacy programs have the potential to improve knowledge and reduce stigma, this may help youth and trained educators distinguish between normal everyday negative experience and a potential mental health problem or mental disorder; and may enable them to seek help needed accurately and appropriately. As such, we hypothesized that these population health outcomes may be closely linked to positive clinical outcomes in mental health care, such as early access to care, accurate identification of cases to various levels of care systems (e.g., primary care, community services, specialized clinics, and emergency and inpatient tertiary care systems), and number of referrals from mental health staff in schools to mental health care. More specifically, the current study was designed to test the following hypothesis: School-based mental health literacy training will affect the frequency and quality of referrals positively, such as global function, strengths/concerns, and clinical urgency and severity (e.g., more urgent and severe cases); demographic variables (age, sex, and family composition) (e.g., earlier identification); system variables (frequency of referral, repeat admissions and emergent or scheduled service level, and referral reasons) (e.g., referrals with more severe cases), as determined by a number of validated tool to evaluate the mental health profile of patients admitted to services in the current study.

In September 2013, a mental health literacy intervention School Mental Health Literacy Improvements for Educators and Students (SMILES) was initiated by the Child and Adolescent Addictions Mental Health and Psychiatry Program (CAAMHPP) in the Calgary Zone of Alberta Health Services (AHS). The CAAMHPP represents regional community and hospital-based child and adolescent mental health services that were integrated in 2001. The three-year project, known by schools as the Mental Health Literacy Project, was funded by the Alberta Children’s Hospital foundation and included training educators in local school boards in the identification and referral of students at risk of mental disorders to the appropriate mental health professionals. School staff were trained through one-day workshops that discussed common mental disorders found in youth, their presentation in the school setting and steps to making effective referrals, as well as strategies to reduce stigma towards mental illness. This paper focuses on the impact of the one of the SMILES training components: the "Go-To Educator" training [16] (described below) with educational professionals and its subsequent impact on student access to mental health services and clinical profiles of students entering those services.

Employing standardized data from blind assessment of referrals to regional child and adolescent mental health services, the effect of “Go-To Educator” training on referral rates and the measured quality of referrals was examined under the hypothesis that school-based mental health literacy would may improve the frequency and quality of referrals.

## Materials and methods

### Intervention

The Go-To Educator intervention was developed in 2009 (https://mhlcurriculum.org/professional-learning/go-educator-professional-learning/). This ’gatekeeper’ type approach recognizes that in every school, there are key people on school staff that students feel more comfortable in going to with problems or concerns. The Go-To Educator intervention uses this already existing school-based ecological framework to enhance the capacity of these school staff to be able to identify young people who may have a mental disorder, link them to school-based student services providers for assessment, triage and referral, if necessary, and to support identified youth in the school setting. This intervention is an essential component of the *School-Based Integrated Pathway to Care Model* developed to have a small number of selected staff in schools being trained in improved identification, familiarity with common mental disorders, and capacity to link students with the health system [22]. Staff members who were initially selected for training were chosen by principals or were in roles that were well situated for identification (i.e. guidance counselors, resource teachers, special education teachers). However, soon after the intervention began, numerous schools decided to expose their entire staff to the intervention workshops. Therefore, to accommodate this demand, workshops were delivered in two formats: ’whole school’ trainings and ’open’ trainings. The trainings were provided at schools on a determined professional development day with the entire staff attending, or it allowed schools from different boards to send delegates to be trained at a health services site. All Go-To trainings were similar in content and format, and were provided by the master trainer or by board designated trainers (Core Trainers). The trainings were 6 hours in length and focused on mental health knowledge identification of those at risk in the school setting, providing students support and linking with parents and health care providers [23].

### Setting

There are 438 schools in the Calgary Health Zone serving approximately 273,400 school-age children during the study period (https://www12.statcan.gc.ca/census-recensement/2016/dp-pd/prof/details/page.cfm). The schools are public (n = 299), secular (n = 115) private (n = 18) and chartered (n = 6). Of these schools, 208 participated in the training over the study period (2013–2016). The regional CAAMHPP consist of a central intake and registration system (RAIS), approximately 100 distinct clinical services that include both community and hospital based ambulatory services as well as mobile emergency services and hospital-based emergency and inpatient services. CAAMHPP-school-based services operated before and during the period of “Go-To Educator” training. Referral practices were relatively stable over the period of study, with school-based mental health workers primarily making referrals to central intake or clinics as was their typical practice.

### Study design

The study was approved by Conjoint Health Research Ethics Board at the University of Calgary (REB14-0554), Canada. This is the secondary analysis with approved access to a provincial health database with regards to diagnosis and treatment of young people.

This naturalistic study employed a retrospective cohort design comparing exposure versus non-exposure, as well as pre- and post-exposure to Go-To Educator training with the outcome being clinical status of referrals measured on presentation to and discharge from CAAMHPP services. The dependent variable consisted of three categories: non-participating schools (Group 1), participating schools pre-training (Group 2), and participating schools post-training (Group 3). Group comparison was based on the routinely collected standardized clinical screening measures, demographics, and system variables of cases referred to mental health services (described below). For the purpose of examining the effects of time Group 1 was also broken into pre- and post- groups (Group1 a & b) on the same timeline of the initiation of the Go-To Educator training.

Over the study period there were 54,158 referrals. The mean number of referrals from any referral source was 27. Where a school ID was recorded, 239 untrained schools averaged 18 referrals, 71 pre-training schools also averaged 18, while post-training 194 schools averaged 43 associated referrals. The number of referrals without identified schools (e.g., other referral sources) increased slightly over the study period. The number of referrals from schools exposed to training increased, and the number of referrals from unexposed schools decreased over the study period. Post-training referrals increased over the study, while pre-training referrals from exposed schools decreased.

### Measures

There were three domains of independent clinical measures collected on referral, admission and discharge: ratings of global function, strengths/concerns, clinical urgency and severity; demographic variables (age, sex, and family composition); and system variables (frequency of referral, repeat admissions and emergent or scheduled service level, and referral reasons). These domains of measures were collected by trained central intake staff who were blind to the participation of the referred cases’ school status in respect to having had the school-based Go-To Educator intervention. Thus, these data served as relatively objective independent measures that were employed to analyze the quality of referrals in addition to referral rates. As such these independent variables are more objective indicators of Go-To Educator training effect on referral practice than are the typical self-report instruments.

Global function was rated using two validated clinical measures [24]: Child Global Assessment Scale (CGAS: A measure out of 100 where lower scores represent greater severity) [25], adapted from the adult Global Assessment Scale [26]; and a strength-concern rating scale: Strength/Concern (SC: Lower scores represent greater severity) based on the Goal Attainment Scaling model of community child and family mental health service evaluation [27]. These two scales are those routinely used by the AHS at patient intake [24]. All other mental health services data were obtained from the validated Western Canada Waitlist Project Children’s Mental Health Priority Criteria Score Form (WCWL-CMH-PCS) [28, 29]. All of these data were collected over the course of referral and admission in Regional Access and Intake System (RAIS), the common database used by the Child and Adolescent Addiction and Mental Health and Psychiatry Program (CAAMHPP). These instruments provided the basis for measuring changes in the mental health clinical profiles (e.g., function, severity, urgency, etc.) of referrals for treatment before and after the Go-To Educator program, in comparison to referrals from untrained schools during the study period.

A total of 41 variables (17 WCWL-CMH-PCS items, 2 CGAS items, 2 SC items, 4 demographics items, 5 system items, and 11 referral reasons items), were used as independent variables to profile referrals made from Go-To Educator training exposed (before and after training) and unexposed schools. Hence, exposed schools, before and after training, as well as unexposed schools represent the three categories of the dependent variable.

For demographic variables we calculated students’ average age admitted to services. For gender ’female’ was used as baseline assigning it the value of "0" and for “male” the value assigned was "1". The closer the value is to 1, the more male admissions. Similarly, for the "family composition" variable we assigned "biological parent/step parent" as the baseline (0) and "single parent or foster care/ward" as 1. This grouping of family composition represents low- and high-risk strata within clinical populations, respectively [30, 31].

For the system variable, "Scheduled" was assigned as the baseline with the value of 0 and "Emergent" was assigned the value of 1. “Reason for referral” was derived from a standard pick-list. Repeat admissions is a variable that sums the number of registrations for a particular patient and number of provisional comorbid diagnoses. Length of stay (LOS) was calculated in days from admission to discharge dates, wait time was calculated in days from referral to admission dates, and repeat admissions were the counts or frequencies of admissions.

### Data collection

Data collection from identified schools occurred between December 2013 and April 2016. Unexposed schools also included referrals from all other sources as all referrals necessarily attend school by law. During this time school training dates were tracked. Students presenting to mental health services during this time were assigned to intervention or comparison groups based on the training status (training received/training not received) of the school they attended at the time of referral. Retrospectively, students that presented to mental health services prior to their school receiving training were placed in the "before training" category. Scores in each of the independent variables permitted comparison of referral quality and quantity post training as compared to pre-training (within-school comparison) and untrained (between-school comparison).

Anonymous data from eligible (designated current school on registration) consecutive referrals was extracted from the regional registration system. Demographic, clinical urgency and severity, function and problem severity data was obtained from WCWL-CMH-PCS [27] and the CGAS [25] and SC scales [24].

### Participants

The sample was drawn from consecutive referrals (n = 54158; 54.86% female; 208 schools) (Table 1) with those in the before and after groups having a current school noted on their registration record in the CAAMHPP program of the AHS, Calgary Health Zone. There were significantly more female than male participants (p < .00001) in the sample. The Go-To Educator training had been designed for secondary school staff who interact with students between the ages of 12–18 years. However, schools in Calgary are broken into primary (Kindergarten to Grade 6: up to age 12 years), junior high (Grades 7–9: ages 13–14 years) and high school (Grades 10–12: ages 15–17 years). Hence, schools with staff from every grade level could be exposed to training.

**Table 1:** Description of independent variables by dependent groups and bivariate multinomial logistic regression of independent variables by dependent groups. All individual WCWL CMH-PCS Item data calculated based on the same number of participants in the total score calculation and therefore were not included in the individual cell.

Variable	No Exposure < 2014 Proportion (LCI, UCI) Odds Ratio (OR) (LCI, UCI) Group 1a	No Exposure > 2013 Proportion (LCI, UCI) Odds Ratio (OR) (LCI, UCI) Group 1b	Not Exposed Admission Before Smiles Proportion (LCI, UCI) Odds Ratio (OR) (LCI, UCI) Group 2	Exposed Admission After Smiles Proportion (LCI, UCI) Odds Ratio (OR) (LCI, UCI) Group 3
**WCWL CMH-PCS Items**				
WCWL CMH-PCS Total Score (1–100)	n = 32,556 41.668 (41.494, 41.842) OR = 1.031 (1.028, 1.034)* ^&^ **	n = 14,974 35.971 (35.791, 36.151) OR = 1.003 (1, 1.006)	n = 423 35.645 (34.646, 36.645) OR = 1.001 (0.993, 1.008)	n = 2,892 35.484 (35.082, 35.886)
1-danger to oneself	2.616 (2.577, 2.655) OR = 1.341 (1.306, 1.378)* ^&^ **	.948 (.92, .976) OR = 1.037 (1.008, 1.067)*	.617 (.553, .681) OR = 0.796 (0.708, 0.896)*	.871 (.82, .921)
2-danger to others	.333 (.327, .34) OR = 2.831 (2.539, 3.157)*^,^ **	.122 (.116, .128) OR = 1.139 (1.015, 1.279)*	.085 (.057, .113) OR = 0.819 (0.588, 1.141)	.106 (.094, .118)
3-psychotic symptoms	.89 (.865, .916) OR = 1.356 (1.298, 1.416)*^,^ **	.234 (.217, .252) OR = 1.048 (1.001, 1.097)*	.199 (.119, .278) OR = 1.004 (0.891, 1.131)	.196 (.162, .229)
4-gloabal age-appropriate developmental progress	.224 (.219, .228) OR = 1.828 (1.638, 2.039)*^,^ **	.141 (.136, .147) OR = 1.044 (0.93, 1.172)	.149 (.115, .183) OR = 1.11 (0.832, 1.48)	.136 (.124, .149)
5- children’s global assessment of function scale (CGAS)	7.702 (7.661, 7.743) OR = 1.07 (1.059, 1.081)*^,^ **	6.988 (6.939, 7.037) OR = 1.011 (1, 1.022)	6.922 (6.637, 7.207) OR = 1.006 (0.978, 1.035)	6.846 (6.736, 6.957)
6- internalized symptoms	7.442 (7.387, 7.498) OR = 1.057 (1.048, 1.065)*^,^ **	6.019 (5.955, 6.082) OR = .99 (0.982, 0.999)*	5.816 (5.471, 6.16) OR = .98 (0.959, 1.003)	6.227 (6.088, 6.366)
7- externalized symptoms/disruptive behavior	1.647 (1.63, 1.664) OR = 1.307 (1.272, 1.343)*^,^ **	1.149 (1.126, 1.172) OR = 1.045 (1.016, 1.076)*	1.026 (.898, 1.154) OR = .981 (0.91, 1.057)	1.062 (1.012, 1.113)
8- comorbid medical conditions	.469 (.461, .478) OR = 1.569 (1.472, 1.672)*^,^ **	.304 (.294, .314) OR = 1.105 (1.033, 1.182)*	.215 (.165, .266) OR = 0.845 (.698, 1.022)	.268 (.246, .29)
9- comorbid psychiatric conditions	1.179 (1.16, 1.198) OR = 1.123 (1.094, 1.152)*^,^ **	.86 (.838, .882) OR = 0.991 (.964, 1.018)	.827 (.711, .944) OR = .975 (.908, 1.048)	.88 (.831, .929)
10- harmful substance use/misuse	.171 (.167, .175) OR = 3.544 (3.012, 4.169)*^,^ **	.062 (.058, .065) OR = 1.128 (.948, 1.341)	.073 (.048, .098) OR = 1.359 (.912, 2.026)	.055 (.047, .063)
11- significant biological family history of mental illness	1.359 (1.349, 1.369) OR = .922 (.884, .962)*	1.397 (1.383, 1.412) OR = .964 (.923, 1.008)	1.362 (1.272, 1.451) OR = .925 (.829, 1.033)	1.427 (1.394, 1.46)
12- school and/or work difficulties	.336 (.331, .341) OR = 1.842 (1.681, 2.018)*^,^ **	.215 (.208, .221) OR = .994 (.902, 1.095)	.21 (.171, .249) OR = .969 (.754, 1.244)	.216 (.201, .231)
13- social/friendships/community functioning	.702 (.697, .707) OR = 1.349 (1.246, 1.461)*^,^ **	.617 (.61, .625) OR = .925 (.852, 1.005)	.622 (.575, .668) OR = .943 (.764, 1.164)	.636 (.618, .653)
14- does the child/adolescent [patient] have problems in the context of the home?	4.014 (3.995, 4.034) OR = 1.218 (1.193, 1.244)*^,^ **	3.402 (3.375, 3.429) OR = 1.006 (.985, 1.028)	3.305 (3.159, 3.451) OR = .978 (.926, 1.033)	3.381 (3.318, 3.444)
15- family functioning or factors affecting child	.706 (.701, .711) OR = 1.561 (1.444, 1.689)*^,^ **	.587 (.579, .595) OR = .925 (.852, 1.003)	.605 (.558, .652) OR = .997 (.81, 1.229)	.606 (.588, .624)
16- prognosis without further intervention	6.45 (6.398, 6.502) OR = 1.042 (1.033, 1.05)*^,^ **	5.904 (5.828, 5.98) OR = 1.017 (1.009, 1.026)*	6.317 (5.864, 6.769) OR = 1.036 (1.014, 1.058)*	5.52 (5.35, 5.69)
17- degree of likely benefit with further intervention	9.035 (9.004, 9.067) OR = 1.108 (1.091, 1.125)*^,^ **	8.365 (8.325, 8.405) OR = 1.012 (.996, 1.028)	8.277 (8.058, 8.495) OR = .997 (.957, 1.039)	8.293 (8.203, 8.382)
**ACE items (1–10)**				
ACE total score (0–10)	n = 3,488 3.611 (3.522, 3.7) OR = 1.047 (1.03, 1.064)*^,^ **	n = 21,559 3.221 (3.185, 3.256) OR = .992 (.98, 1.004)	n = 457 3.575 (3.326, 3.825) OR = 1.042 (1.006, 1.08)*	n = 4,523 3.279 (3.204, 3.354)
1-Physical abuse	n = 3,812 .506 (.49, .522) OR = 1.195 (1.097, 1.301)*^,^ **	n = 22,643 .425 (.419, .432) OR = .863 (.811, .92)*^,^ **	n = 476 .468 (.423, .514) OR = 1.029 (.852, 1.243)	n = 4,703 .461 (.447, .476)
2-Sexual abuse	n = 3,743 .201 (.188, .214) OR = 1.186 (1.078, 1.305)*	n = 22,523 .157 (.152, .161) OR = .925 (.861, .994)*	n = 477 .201 (.166, .24) OR = 1.015 (.819, 1.257)	n = 4,682 .15 (.139, .16)
3-Emotional abuse	n = 3,833 .513 (.497, .529) OR = 1.43 (1.277, 1.602)*^,^ **	n = 22,661 .487 (.48, .493) OR = 1.057 (.968, 1.154)	n = 475 .48 (.434, .526) OR = 1.433 (1.13, 1.818)*	n = 4,701 .511 (.497, .526)
4-Physical neglect	n = 3,848 .22 (.207, .234) OR = 1.008 (.926, 1.098)	n = 22,711 .19 (.185, .195) OR = .906 (.851, .965)*	n = 480 .19 (.155, .228) OR = .883 (.731, 1.066)	n = 4,721 .187 (.176, .198)
5-Emotional neglect	n = 3,853 .64 (.625, .655) OR = 1.229 (1.105, 1.366)*	n = 22,710 .55 (.544, .557) OR = 1.02 (.941, 1.105)	n = 481 .651 (.606, .693) OR = 1.017 (.8, 1.292)	n = 4,741 .561 (.547, .575)
6-Exposure to domestic violence	n = 3,691 .291 (.277, .306) OR = 1.393 (1.276, 1.52)*^,^ **	n = 22,273 .292 (.286, .298) OR = .957 (.899, 1.019)	n = 472 .301 (.26, .344) OR = 1.458 (1.198, 1.773)*	n = 4,682 .281 (.268, .294)
7-Household substance abuse	n = 3,773 .404 (.388, .42) OR = 1.052 (.956, 1.158)	n = 22,504 .347 (.34, .353) OR = 1.056 (.985, 1.133)	n = 474 .407 (.363, .453) OR = 1.102 (.896, 1.355)	n = 4,687 .36 (.346, .374)
8-Household mental illness	n = 3,750 .672 (.657, .687) OR = 1.203 (1.101, 1.314)*	n = 22,429 .645 (.639, .651) OR = .942 (.883, 1.006)	n = 477 .706 (.663, .747) OR = 1.22 (1.006, 1.48)*	n = 4,705 .655 (.641, .668)
9-Parental separation or divorce	n = 3,871 .145 (.134, .157) OR = 1.081 (.987, 1.184)	n = 22,816 .13 (.126, .135) OR = .957 (.896, 1.023)	n = 481 .179 (.146, .216) OR = 1.269 (1.033, 1.559)*	n = 4,746 .123 (.114, .133)
10-Incarcerated household member	OR = 1.208 (1.067, 1.368)*	OR = 1.064 (0.968, 1.17)	OR = 1.546 (1.205, 1.982)*	
**Demographic/System Variables**				
High Risk Family Composition (Compared to Low Risk)	n = 30,417 .41 (.405, .416) OR = 1.034 (.973, 1.1)	n = 23303 .413 (.407, .42) OR = 1.047 (.984, 1.115)	n = 624 .465 (.425, .505) OR = 1.29 (1.091, 1.525)*	n = 4944 .402 (.389, .416)
High Risk Sex Category	n = 47,912 .508 (.504, .513) OR = .71 (.673, .749)*^,^ **	n = 41,521 .574 (.57, .579) OR = .927 (.878, .978)*	n = 768 .581 (.545, .616) OR = .951 (.817, 1.108)	n = 6,238 .593 (.581, .605)
Emergency/Inpatient (Compared to Scheduled Admissions)	n = 47,912 .31 (.306, .314) OR = 1.03 (.97, 1.09)	n = 41,521 .295 (.29, .299) OR = .955 (.9, 1.01)	n = 768 .258 (.227, .29) OR = .795 (.67, .94)*	n = 6,238 .304 (.293, .316)
Age (Years)	n = 47,912 12.637 (12.596, 12.678) OR = .893 (.886, .899)*^,^ **	n = 41,521 13.159 (13.116, 13.203) OR = .916 (.909, .922)*^,^ **	n = 768 13.69 (13.452, 13.928) OR = .943 (.926, .961)*^,^**	n = 6,238 14.568 (14.503, 14.632)
Number of Registrations	n = 47,912 5.561 (5.511, 5.611) OR = .971 (.967, .975)*^,^ **	n = 41,521 6.13 (6.076, 6.184) OR = .989 (.985, .993)*^,^ **	n = 768 6.004 (5.643, 6.365) OR = .985 (.973, .998)*	n = 6,238 6.555 (6.408, 6.702)
Overall length of stay (from index to last admission)	n = 47,890 1135.181 (1124.687, 1145.675) OR = 1.0002 (1.0002, 1.0002)*^,^**	n = 38,526 871.676 (861.723, 881.629) OR = 1 (0.9999, 1)*	n = 755 989.446 (915.179, 1063.714) OR = 1.0001 (1, 1.0001)	n = 5,992 916.27 (891.346, 941.194)
Index Admission Problem Severity	n = 26,861 2.314 (2.298, 2.331) OR = .827 (.812, .842)*^,^ **	n = 29,627 2.742 (2.723, 2.762) OR = .996 (.979, 1.013)	n = 652 2.772 (2.66, 2.885) OR = 1.006 (.959, 1.055)	n = 5,229 2.755 (2.714, 2.795)
Index Admission CGAS (Function)	n = 42,145 41.507 (41.381, 41.633) OR = 1.014 (1.011, 1.016)*^,^ **	n = 35,869 39.692 (39.554, 39.829) OR = 1.003 (1.001, 1.005)*	n = 747 41.345 (40.512, 42.178) OR = 1.013 (1.007, 1.018)*	n = 5,986 39.175 (38.851, 39.499)
**Reasons for Referral (Proportions in Relation to Externalizing Issues)**				
Internalizing/Emotional issues	n = 38,211 .327 (.322, .332) OR = .348 (.317, .381)*^,^**	n = 22,273 .304 (.299, .308) OR = 0.462 (.421, .507)*^,^**	n = 472 .482 (.446, .518) OR = .782 (.618, .99)*	n = 4,682 .454 (.442, .467)
Developmental/ organic concerns	n = 38,211 .019 (.018, .021) OR = 1.609 (1.14, 2.27)*	n = 22,504 .017 (.016, .019) OR = 2.071 (1.467, 2.923)*^,^**	n = 474 .009 (.004, .019) OR = 1.165 (.505, 2.687)	n = 4,687 .006 (.004, .008)
Eating issues	n = 38,211 .041 (.039, .043) OR = 1.51 (1.192, 1.913)*	n = 22,429 .019 (.018, .02) OR = 1.002 (.787, 1.276)	n = 477 .016 (.008, .027) OR = 0.877 (.462, 1.664)	n = 4705 .013 (.01, .016)
Adjustment problems	n = 38,211 .025 (.024, .027) OR = .605 (.494, .741)*^,^**	n = 22,816 .018 (.017, .02) OR = .628 (.511, .772)*^,^**	n = 481 .025 (.015, .038) OR = .903 (.534, 1.528)	n = 4,746 .02 (.017, .024)
School/Learning/Attention problems	n = 38,211 .039 (.037, .041) OR = .879 (.723, 1.068)	n = 23,303 .023 (.022, .025) OR = .76 (.623, .927)*	n = 624 .029 (.018, .043) OR = .991 (.603, 1.629)	n = 4,944 .021 (.018, .025)
Social/Family issues	n = 38,211 .068 (.066, .071) OR = 1.147 (.965, 1.362)	n = 41,521 .058 (.056, .061) OR = 1.406 (1.183, 1.672)*^,^**	n = 768 .022 (.013, .035) OR = .569 (.332, .976)*	n = 6,238 .029 (.025, .033)
Thought disturbances/Perceptual issues	n = 38,211 .027 (.025, .028) OR = .837 (.668, 1.048)	n = 41,521 .019 (.018, .021) OR = .873 (.696, 1.096)	n = 768 .02 (.011, .032) OR = .936 (.523, 1.676)	n = 6,238 .015 (.012, .019)
Harmful behavior/thoughts to Self	n = 38,211 .178 (.175, .182) OR = .337 (.305, .372)*^,^**	n = 41,521 .234 (.23, .238) OR = .63 (.571, .696) *^,^**	n = 768 .202 (.174, .232) OR = .581 (.446, .759)*^,^**	n = 6,238 .256 (.245, .267)
Harmful behavior/thoughts to Others	n = 38,211 .015 (.014, .016) OR = .29 (.239, .353)*^,^**	n = 41,521 .024 (.023, .026) OR = .671 (.556, .81)*^,^**	n = 768 .013 (.006, .024) OR = .384 (.196, .752)*	n = 6,238 .025 (.021, .029)
Addictive or Legal issues	n = 38,211 .011 (.01, .012) OR = .438 (.338, .567)*^,^**	n = 38526 .015 (.013, .016) OR = .837 (.649, 1.078)	n = 755 .01 (.005, .02) OR = .639 (.299, 1.364)	n = 5,992 0.012 (.009, .015)
Other	n = 38,211 .045 (.043, .047) OR = .441 (.381, .511)*^,^**	n = 29,627 .125 (.121, .128) OR = 1.742 (1.512, 2.007)*^,^**	n = 652 .039 (.027, .055) OR = .583 (.38, .896)*	n = 5229 .049 (.044, .055)

* represents p<0.05;

** represents p<0.001.

Odds ratio were calculated through Bivariate Multinomial Logistic Regression of Independent Variables by Dependent Groups, using “Exposed Admission After Smiles” group as the comparison group.

### Data analysis

We examined the clinical profiles of three groups based on the quality of referrals (blind third-party clinical measures) that included referrals from exposed schools before training, exposed schools after training and un-exposed schools. The referral rates from the three groups (untrained schools (group 1 a & b), before training (Group 2) and after training schools (Group 3) were also compared. To examine the effect of time and exposure, the unexposed schools were broken into two pre- and post- groups roughly corresponding to the pre-post period of the exposed schools. In bivariate analysis, the independent variables were compared between the three referral groupings (dependent variable) on the basis of overlapping versus non-overlapping 95% confidence intervals with z-score set to 1.96 indicated un-biased significant differences. Additionally, logistic regression analysis was conducted to identify the most important variables predicting membership in each of the groups with the post-training group serving as the baseline comparison. Demographic variables were included as covariates of analysis.

## Results

Table 1 describes the sample for each of the dependent variable groups across all independent variables and provides both the 95% CIs for each group for the purpose of comparison. Table 1 further presents the results of bivariate multinomial logistic analysis noting the magnitude of the significant differences. Odds ratios greater than the value 1 indicate that the likelihood of group membership is greater in the indicated group as compared to the base group representing admissions after Go-To Educator intervention Exposure. Of the forty-eight independent variables, there were eighty-two significant differences at p < .05 across the three groups in comparison to exposure to the Go-To Educator training before admission group. Forty-six differences were significant at p < .0001 across the three groups in comparison to exposure to the Go-To Educator training before admission group.

Table 2 presents the results of three logistic analyses comparing each group separately to the base group: Go-To Educator invention exposure before admission. Each was analyzed separately to overcome the effects of potential multicollinearity, due to the fact that all variables are measuring some aspect of clinical psychopathology.

**Table 2:** Estimates comparing admissions after smiles exposure to all other groups in three logistic models.

Compared to Smiles Exposure Before Admission (Value 0) Group 2	Smiles Exposure After Admissions (Value 1) Group 3	No Exposure Admission<2014 (Value 1) Group 1a	No Exposure Admission>2013 (Value 1) Group 1b
ACE item: Physical neglect	0.499**		
WCWL Item: FAMILY FUNCTIONING OR F~F	0.493**		
WCWL Item: EXTERNALIZED SYMPTOMS/D~E	0.317***		
WCWL Item: SIGN IFICANT BIOLOGICAL~H	0.287***		
WCWL Item: DANGER TO SELF	0.272**		
WCWL Item: INTERNALIZED SYMPTOMS	0.111***		
Age	0.093***		
Length of Stay from Index to Last Admission	.0001***		
CGAS on Index Admission	-0.015*		
Total WCWL score on Index Admission	-0.071***		
High Risk Sex (Female/Self-Defined)	-0.421**		
ACE item: Incarcerated household relative	-0.501*		
ACE item: Exposure to domestic violence	-0.962***		
Constant	3.128***		
Referral Reason: Adjustment problems		0.452*	
WCWL Item: SOCIAL/FRIENDSHIPS/COMM~N		0.160*	
Referral Reason: Internalizing/Emotional issues		0.158*	
Age		0.106***	
WCWL Item: EXTERNALIZED SYMPTOMS/D~E		0.101***	
Problem Severity on Index Admission		0.080***	
WCWL Item: DANGER TO SELF		0.045*	
WCWL Item: INTERNALIZED SYMPTOMS		0.030**	
Length of Stay from Index to Last Admission		-0.0001***	
Number of Admissions		-0.014**	
Total WCWL score on Index Admission		-0.016***	
Emergency/Inpatient Admission		-0.211**	
Referral Reason: Eating issues		-0.574*	
Referral Reason: Other		-1.047***	
Constant		-2.420***	
ACE item: Physical neglect			1.053***
ACE item: Parental separation or ~e			0.973***
ACE item: Household substance abuse			0.826***
ACE item: Emotional neglect			0.767***
ACE item: Exposure to domestic vi~e			0.681***
ACE item: Sexual abuse			0.609***
Age			0.477***
ACE item: Household mental illness			0.458**
ACE item: Emotional abuse			0.457**
ACE item: Incarcerated household ~r			0.422*
WCWL Item: SCHOOL AND/OR WORK			0.396**
WCWL Item: SOCIAL/FRIENDSHIPS/COMM~N			0.384**
WCWL Item: EXTERNALIZED SYMPTOMS/D~E			0.269***
Problem Severity on Index Admission			0.250***
Total WCWL score on Index Admission			0.066***
Length of Stay from Index to Last Admission			-0.001***
WCWL Item: INTERNALIZED SYMPTOMS			-0.079***
WCWL Item: PROGNOSIS WITHOUT FURTH~V			-0.088***
WCWL Item: CHILDREN’S GAF (CGAS) G~S			-0.123***
WCWL Item: DEGREE OF LIKELY BENEFI~U			-0.135***
WCWL Item: CO-MORBID MEDICAL CONDI~S			-0.177*
WCWL Item: PSYCHOTIC SYMPTOMS			-0.205***
WCWL Item: CO-MORBID PSYCHIATRIC C~S			-0.206***
WCWL Item: DANGER TO SELF			-0.255***
High Risk Sex (Female/Self-Defined)			-0.583***
Total ACE score on Index Admission			-0.649***
Emergency/Inpatient compared to Scheduled Admission			-0.749***
Referral Reason: Other			-0.893***
WCWL Item: HARMFUL SUBSTANCE USE/M~E			-0.933***
Referral Reason: School/Learning/Attention problems			-0.951***
Referral Reason: Eating issues			-1.321**
Constant			-2.946***
Sample Size	2035	3668	8070
Model p <	0.00001	0.00001	0.00001

* p<0.05;

** p<0.01;

*** p<0.001

Compared to smiles exposure before admission, smiles exposure after admission was increased in the following variables: ace item: physical neglect; WCWL items: 15-family functioning or factors affecting child, 7-externalized symptoms/disruptive behavior, 11-significant biological family history of mental illness, 1-danger to self, 6-internalized symptoms, age, and length of stay from index to last admission.

Compared to smiles exposure before admission, smiles exposure after admission was reduced in the following variables: CGAS on index admission, total WCWL score on index admission, high risk sex (female/self-defined) ace items incarcerated household relative and exposure to domestic violence.

Compared to smiles exposure before admission, no exposure admission before 2014 was increased in the following variables: referral reason: adjustment problems, WCWL items 13-social/friendships/community functioning, 7-externalized symptoms/disruptive behavior, 1-danger to self, 6- internalized symptoms, as well as problem severity on index admission referral reason internalizing/emotional issues and age.

Compared to smiles exposure before admission, no exposure admission before 2014 was reduced in the following variables: length of stay from index to last admission, number of admissions, total WCWL score on index admission, emergency/inpatient admission and referral reasons eating issues and other.

Compared to smiles exposure before admission, no exposure admission after 2013 was increased in the following variables: ace item: physical neglect, parental separation or divorce, household substance abuse, emotional neglect, exposure to domestic violence, sexual abuse, household mental illness, emotional abuse, and incarcerated household member and in WCWL items 12- school and/or work difficulties, 13-social/friendships/community functioning, 7-externalized symptoms/disruptive behavior, as well as problem severity on index admission, total WCWL score on index admission and age.

Compared to smiles exposure before admission, no exposure admission after 2013 was reduced in the following variables: length of stay from index to last admission, WCWL items 6-internalized symptoms, 16-prognosis without further intervention, 5-children’s global assessment of function scale (CGAS), 17-degree of likely benefit with further intervention, 8-comorbid medical conditions, psychotic symptoms, 9-comorbid psychiatric conditions, danger to self, harmful substance use/misuse as well as high risk sex (female/self-defined), total ace score on index admission, emergency/inpatient compared to scheduled admission and referral reasons school/learning/attention problems, eating issues and other.

## Discussion

The results of this study show evidence that access to care may be influenced by specialized training of educational staff. These employed methods similar to another study focusing on mental health literacy in primary care [32]. In general, the Go-To Educator training significantly changed the demographics, clinical profile, and treatment effects of students presenting to tertiary mental health services and substantially increased the referral rate of associated cases compared to untrained schools and schools pre-training. Changes were observed in respect to referral reasons, demographic characteristics (e.g., age, sex, and family compositions), clinical profiles (e.g., comorbid psychiatric diagnoses, danger to self/others, development progress, externalized symptoms, substance use, problems at home), most of the system variables (e.g., length of stay, repeat admissions), and some treatment effect variables (e.g., admission/discharge strength concern). Therefore, mental health literacy training in schools had a contextual effect on a substantial number of the independent variables compared to the non-exposed groups and pre-group. Taken together, the results are consistent with the mandate of mental health literacy training in schools, indicating that mental health literacy training in schools appears to improve identification of students generally with greater clinical severity and therefore more in need of care.

Findings in this study are responses to the call for school-based mental health programs by Mental Health Commission of Canada [33] to effectively address early identification of mental health problems or mental disorders among youth. While research suggests that the system-level conditions are essential for school mental health programs to flourish [34], there are system-level gaps such as protocol and agreements defining the pathway to services in the school community and insufficient professional development for school staff [35]. This study built a mechanism for education and health systems to initiate the dialogue and presented an example how between-system collaborations may be established by sharing data and therefore to facilitate the pathway through care.

Changes in referral reasons are in good alignment with how Go-To Educators were trained to identify at-risk students. For example, students referred to services from after-training schools were more likely to demonstrate adjustment problems, thought disturbances, and harmful behaviours/thoughts to others, and this may be because trained teachers had realized these challenges were related to potential mental disorders, such as anxiety or psychosis, rather than behavioural challenges alone. Meanwhile, we observed fewer students reported as having danger to self at intake, possibly because students were being sent for services before becoming suicidal or engaging in problematic self-harm behaviours. We found fewer students presenting to tertiary services for social/family issues perhaps because the Go-To Educator training taught that most of these challenges can be dealt with in the community without need to access tertiary care mental health services.

In addition, developmental concerns were no longer major referral reasons compared to no-training schools. This may have resulted because developmental concerns were not a key focus of the Go-To Educator training, which addresses mental disorders commonly start during adolescence such as anxiety, Attention Deficit Hyperactivity Disorder, Depression, and Schizophrenia. Eating disorders were less identified as well because the training effectively taught how to distinguish between normal dieting patterns and eating disorders so that Go-To Educators obtained the capacity for appropriate referral. Similarly, the number of students referred to services for addiction or legal issues dropped as a function of the training given; Go-To Educators were directed to refer students with a substance use disorder to Youth Addiction Services who do not currently share the same registration system with the rest of Child and Adolescent Services. This may have impacted the specificity of the referral.

Further, based on the fact that students referred to services were younger we hypothesized that the Go-To Educator training had improved early identification capacity among educators in the after-training schools. Identified students were also more from single parent family, indicating that educators may have become more sensitized to the needs of single parent families for mental health support.

We also found that although students from the after-training schools waited longer to be admitted to services, once in care they had more repeat admissions and they stayed longer in the service. These factors speak to the complexity and severity of these cases and the level of required services these students required. The more complex the cases, the more admissions and the longer duration of care provided. These proxy measures thus may suggest that the students referred as a result of the Go-To Educator training had more complex mental health care needs than those referred from control sites. While their symptoms were more severe from after-training schools at admission, the symptom severity was reduced significantly at discharge, meaning treatment was effective helping students to services. These are very encouraging results, implying not only the quality of referral was improved, but also students in need of care received appropriate care.

It should be highlighted that the Go-To trainings were open to any professional affiliated with a school. This included support staff, school-based nurses, occupational therapists, family school liaison workers and school-based police resource officers, in addition to the teachers. Creating a larger shared literacy regarding mental health may have had important impacts on the referral number and profile and is worth further investigation. As such, the educational system may consider to further stabilize the Go-To Educator training in the school setting, including deciding the number of Go-To Educators and number of training sessions in each school, establishing a coordinator role to facilitate the training in each school board, securing consistent funding and linking with the local mental health services. Therefore, the Go-To training intervention may facilitate the links between health and education to effectively address adolescent mental health needs in a collaborative manner in order to sustain the impacts of Go-To training in the long term. This goal may be achieved through the collaborative work by policy makers in both education and mental health in the future.

While an economic analysis was not conducted as part of this study, given the findings presented herein, there may be significant frugalities realized with this approach compared to usual school—clinic referral patterns. The cost of project implementation was minimal—the master trainer had his costs covered by the mental health service. The project formed training teams out of volunteers from health and education to lead the workshops, no additional funds to cover participant time were used for this purpose. Infrastructure costs of the program were negligible, comprising mostly part-time administrative support. The vast majority of trainings occurred on time designated to support existing professional development creating minimal need for substitute teachers to “back-fill” for educator participants. ’School hosted’ trainings eliminated the need for travel for participants, and site training costs such as space rental were covered by the schools in which the training occurred. However, these preliminary cost considerations now need to be appropriately calculated using appropriate economic analysis and will be addressed in future studies [36].

Limitations included the possibility that trained Go-To Educators might move between schools. While such migrations happened, this was not the majority of trained teachers, who were equally likely to migrate to other exposed schools. Students with identified mental problems could also move between schools. Both forms of migration would bias the results in favor of the null hypothesis. Some students with mental problems may have sought assistance from private services (not CAAMHPP), which would similarly bias the results in favor of the null hypothesis.

## Conclusion

To summarize, the Go-To Educator training was shown to be effective to potentially help educators early identify students at risk of mental disorders, accurately distinguish the difference between a mental disorder and everyday challenges or problems, understand different functions of health agencies for appropriate referral of at-risk students, and to support the health system to effectively treat students in need of care. Previous research on mental health training of Canadian educators has been focusing on knowledge improvement, stigma reduction or enhanced help-seeking [14, 17, 37], or the training of in-school mental health professionals (e.g. guidance counsellors, social workers, and health nurses) to address mental health needs of young people in the setting. Our approach to blend teachers and in-school mental health professionals is, to our knowledge, the first of its kind to closely effectively link education and health together and has the potential to reduce the burden of youth mental illness through early identification and improved quality of referral and treatment. This is also the first study using relatively objective independent measures, rather than self-report measures, to evaluate the impact of community based mental health literacy interventions on the mental health system, which may provide information on how to evaluate such interventions in the future.

Staff migration between schools during the training period may have impacted their knowledge of mental health and referrals. Such migration would have dampened the impacts in trained schools and enhanced in untrained schools. Other mental health initiatives that were not coordinated with the Go-To Educator training and were running concurrently may have created impacts in the referral profiles (YAS outreach and Headstrong events). As these were also being applied in comparison schools the training showed impact in spite of this potential confound. Future studies will need to better control for concurrent mental health related activities that may influence referral rate and quality. The study is not a randomized controlled trial design which may lead to biased results due to the potential different demographics of students referred to the services between the exposed and unexposed schools.
